# Association between the adherence to Mediterranean diet and depression in rheumatoid arthritis patients: a cross-sectional study from the NHANES database

**DOI:** 10.1186/s41043-024-00572-w

**Published:** 2024-07-05

**Authors:** Liya Ma, Jingman Yuan, Xichao Yang, Meixi Yan, Ying Li, Min Niu

**Affiliations:** 1 Department of Rheumatology Immunology and Endocrinology, Honghui Hospital Affiliated of Xi’an Jiaotong University, No.76 Nanguo Road, Beilin District, Xi’an, Shaanxi Province 710061 China; 2 Department of Geriatric, Xi’an Qinhuang Hospital, Xi’an, Shaanxi Province 710061 China

**Keywords:** Adherence to mediterranean diet, Depression, Rheumatoid arthritis, NHANES database

## Abstract

**Background:**

Rheumatoid arthritis (RA) is a systemic autoimmune disease, and depression is a most frequent comorbid condition associated with RA. Studies have shown that inflammation plays a vital role in the pathophysiology of depression and RA. Mediterranean diet (MED) has been proved to be a healthy anti-inflammatory dietary pattern. This study aims to explore the association between the adherence to Mediterranean diet (aMED) and depression in RA patients.

**Methods:**

In this study, RA patients aged ≥ 20 years old were extracted from the National Health and Nutrition Examination Survey (NAHNES) database. Dietary intake information was obtained from 24-h dietary recall interview. Covariates included sociodemographic information, lifestyles, laboratory parameters, and the history of diseases and medications were included. The weighted univariable and multivariable logistic regression models were used to assess the association between aMED and depression. Subgroup analysis was conducted to further explore the association between MED components and depression.

**Results:**

Totally 1,148 patients were included, of whom 290 (25.26%) had depression. After adjusted all covariates, high aMED was associated with the lower odds of depression in RA patients (OR = 0.53, 95%CI: 0.29–0.97). Among MED components, higher consumption of vegetables (OR = 0.54, 95%CI: 0.34–0.84) and cereals (OR = 0.63, 95%CI: 0.39–0.99) contributed more to decrease the odds of depression.

**Conclusion:**

Greater aMED may have potential benefits for improving mental health in RA patients. Future large-scale cohort studies are needed to explore the association between aMED and depression in RA patients.

**Supplementary Information:**

The online version contains supplementary material available at 10.1186/s41043-024-00572-w.

## Background

Rheumatoid arthritis (RA) is a common systemic and autoimmune disease with erosive and symmetrical arthritis as the main clinical manifestations [1]. RA severely reduces the quality of life of patients, making it easy for patients to change their mental state while suffering from the pain of decline in physical function caused by joint damage. With the continuous development of the modern “bio-psycho-social” medical model, researchers have paid more attention to the emotional changes of patients with physical diseases [2]. Depression is featured by significant and persistent low mood symptoms clinically and is a common and growing mental health issue worldwide related to considerably diminished role-functioning and quality of life, medical comorbidity and mortality [3]. One meta-analysis reported that 16.8% of RA patients had a severe depressive, 2–3 times more than the general population [4]. In addition, the incidence of depression in people with RA may be higher than observed in diabetes and Parkinson’s disease, as well as other chronic inflammatory diseases [5]. Identifying modifiable factors contributing to depression in RA are important to reduce the burden of RA.

Previous evidence supports that pro-inflammatory cytokines mediate the association between RA and depression [6]. Depression can activate inflammatory factors, aggravate pain in patients with RA, and further aggravate depressive symptoms, forming a vicious cycle [6, 7]. Controlling systemic inflammation levels may be beneficial in reducing depression and burden in patients with RA. Diet is an important influencing factor of RA, and multiple nutritional therapies aimed at anti-inflammatory effects have been enacted for the prevention and control of RA [8]. Adherence of Mediterranean dietary (aMED) score is a commonly used dietary evaluation tool internationally and has been shown to be associated with the level of circulatory inflammation [9–11]. Among the components of the Mediterranean diet (MED), especially omega-3s, antioxidants and dietary fiber may help suppress inflammation in the body through different mechanisms [12, 13]. Previous studies suggest that the use of a MED was beneficial in reducing pain and increasing physical function in people with RA [14, 15]. In addition, several cohort studies have found that greater aMED was associated with a lower risk of depression [16, 17]. However, the association between aMED and depression among RA patients remains unclear. Herein, we conducted the current study to explore the association between aMED and the risk of depression among RA patients using a cross-sectional population in the U.S.

## Methods

### Study design and participants

Data for this study were extracted from the National Health and Nutrition Examination Surveys (NHANES) database (2005–2018) [18]. NHANES is a major survey conducted by the National Centers for Health Statistics (NCHS), a part of Disease Control and Prevention (CDC) and is responsible for the compiling life and health statistics. The requirement of ethical approval for this was waived by the Institutional Review Board of Honghui Hospital Affiliated of Xi’an Jiaotong University, because the data was accessed from NAHNES (a publicly available database). The need for written informed consent was waived by the Institutional Review Board of Honghui Hospital Affiliated of Xi’an Jiaotong University due to retrospective nature of the study.

The inclusion criteria were: (1) patients aged ≥ 20 years old; (2) patients diagnosed as RA; (3) patients with the depression diagnosis; (4) patients with the complete information on dietary intake. The exclusion criteria were: (1) patients with extreme energy intake (male: <500 kcal or > 8000 kcal; women: <500 kcal or > 5000 kcal); (2) missing the data of important covariates [white blood cell (WBC), body mass index (BMI), energy, serum cotinine, neutrophils count and lymphocyte count].

### Depression assessment

The outcome variable was depression diagnosed by a scale. NHANES assessed depressive symptoms in study subjects using the Patient Health Questionnaire Depression Scale (PHQ-9) with 9 questions [19]. Each question in PHQ-9 has 4 options, assigned a score of 0–3, 0 (never), 1 (a few days), 2 (more than a week), and 3 (almost every day). The lowest score of the PHQ-9 scale is 0 points and the highest score is 27 points. Subjects with 0–9 score was classified as normal population, and subjects with 10–27 was classified as depression patients.

### The aMED assessment

Dietary intake information was obtained by 24-h dietary recall interview, which conducted through face-to-face communication at the Mobile Examination Center (MEC). Participants were asked to recall all the type and amount of food and drink consumed in the 24 h preceding the interview, from midnight to midnight, while dietary supplement use was also recorded. aMED score was calculated by two steps [20]. First, 24 h dietary recall data was linked to the United States Department of Agriculture (USDA) Food Pattern Equivalence Database to convert food and beverage intake into USDA equivalent food pattern ingredients. All dietary intake data from the 24-h dietary recall review were taken as dietary intake for each participant. The aMED score (total score = 18) are derived by an assigned value of “0”, “1”, or “2” across nine food categories (vegetables, legumes, fruits, nuts, whole grains, red and processed meats, fish, alcohol and olive oil), with higher scores indicating better adherence to Mediterranean diet pattern.

### Potential covariates

The confounding factors in this study included age (years), gender (male, female), race (Mexican American, other Hispanic, Non-Hispanic White, Non-Hispanic Black, other race), marital status (married, single, unknown), physical activity, and poverty-to-income ratio (PIR). PIR was categorized as < 1.0 (insufficient income), ≥ 1.0 (sufficient income) and unknown [21].

The medical history data adopted in this study were determined on the basis of the medical condition questionnaire (MCQ). Diabetes was defined as fasting blood-glucose ≥ 7.0 mmol/L, hemoglobin A1C (HbAlc) ≥ 6.5%, self-reported diabetes or receiving hypoglycemic therapy. Hypertension was defined as systolic blood pressure (SBP) ≥ 140 mmHg, diastolic blood pressure (DBP) ≥ 90 mmHg, self-reported hypertension or using of antihypertensive drugs. Dyslipidemia was defined as total cholesterol (TC) ≥ 200 mg/dL (5.2 mmol/L), triglyceride (TG) ≥ 150 mg/dL (1.7mmol/L), low density lipoprotein cholesterol (LDL-C) ≥ 130 mg/dL (3.4 mmol/L), high density lipoprotein cholesterol (HDL-C) ≤ 40 mg/dL (1.0 mmol/L), self-reported hypercholesterolemia or receiving fat lowering treatment. Smoking was defined as never smoker (smoking less than 100 cigarettes in lifetime), former smoker (smoking more than 100 cigarettes in life but quit now) and current smoker (smoking more than 100 cigarettes in lifetime and still smoking now). Drinking (yes/no) was measured by the question of “Had at least 12 alcohols drinks/1 years” [22]. Physical activity was expressed as the metabolic equivalent task (MET) and calculated as follows: physical activity (met·min/week) = recommended MET × exercise time for corresponding activities (min/day) × the number of exercise days per week (day) [23]. Medication was identified based on participants’ self-reported use of the following drugs: antirheumatics, nonsteroidal anti-inflammatory agents, glucocorticoids, antidepressants and antipsychotics. Immunologic agents were drugs that can enhancing or suppressing the immune system by modifying the immune response [24]. Immunologic agents (yes/no) were determined with the use of category ID-254 of Prescription Medications_Drug Information (RXQ_DRUG) in the NHANES database.

### Statistical analysis

All statistical analyses were performed by SAS 9.4 (SAS Institute Inc., Cary, NC, USA). Using the proc surveyfreq in SAS software, the final sample size was weighted with SDMVPSU, SDMVSTRA and WTMEC2YR. SDMVPSU means that the masked variance unit pseudo-stratum is sdmvstra, and the masked variance unit pseudo-primary sampling unit (PSU) is sdmvpsu. SDMVSTRA refers to the confidence interval (CI) being applied to assess the reliability of an estimate. WTMEC2YR is the MEC exam weight (wtmec2 year) used for weighting.

Continuous data were expressed as mean and standard error (S.E.), and the weighted t-test was used for comparison between groups. Categorical variables were described as the number and percentage [n (%)], and comparisons between groups used the weighted χ^2^ test. The aMED scores were categorized into quartiles. Univariate logistics regression model was utilized to screen for covariates related to the risk of depression among RA patients, as shown in Table S1. Weighted multivariate logistic regression models were performed to explore the association between aMED and depression among RA patients, with odds ratios (ORs) and 95% CIs. Post-hot test was utilized to verify the differences between different aMED score groups (Table S2 and Table S3). Model I was the crude model without adjusting covariates. Model II adjusted race, smoking, PIR, diabetes, antipsychotics, antidepressants, serum cotinine and neutrophils count. Two-sided *P* < 0.05 was regarded as statistically significant.

## Results

### Characteristics of RA patients

**Fig. 1:**
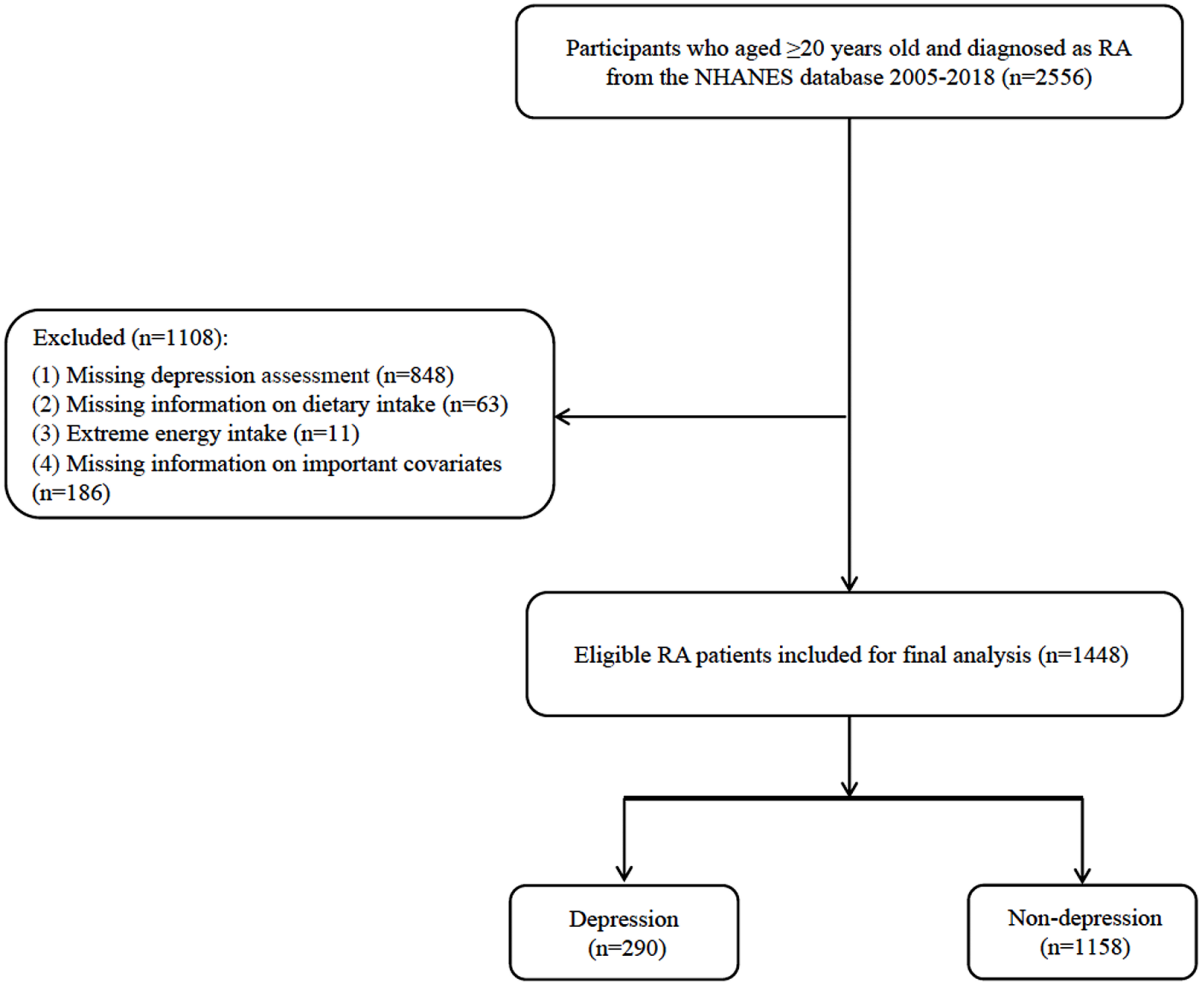
The flow chart of population screening

The screening process for participants in this study was shown in Fig. 1. Totally 2,556 RA patients were screened. Among them, 848 patients missing depression test information, 63 patients missing dietary intake information, 11 patients with extreme energy intake, 40 patients missing WBC data, 21 patients missing BMI data, 107 patients missing energy consumption data, 15 patients missing serum cotinine data, and 3 patients missing neutrophils count were excluded. Finally, 1,448 eligible RA patients were included with the mean age of 59.11 (0.42) years. Of whom, 290 (20.03%) had depression. Characteristics of included participants were shown in Table 1. The proportion of high aMED in non-depression group was significantly higher than in depression group (40.18% vs. 22.96%). Differences were found in race, segmented neutrophils, smoking, the level of PIR, WBC, serum cotinine and aMED, the history of diabetes and the history of antidepressants and antipsychotics using between two groups (all *P* < 0.05).

**Table 1:** Characteristic of RA patients

Variables	Total (*n* = 1448)	Non-depression (*n* = 1158)	Depression (*n* = 290)	Statistics	*P*
Age, years, Mean (S.E)	59.11 (0.42)	59.43 (0.49)	57.56 (0.85)	t = 1.82	0.071
Gender, n (%)				χ^2^ = 1.973	0.160
Male	522 (35.41)	435 (36.60)	87 (29.79)		
Female	926 (64.60)	723 (63.40)	203 (70.21)		
Race/Ethnicity, n (%)				χ^2^ = 18.886	< 0.001
Mexican American	148 (4.51)	97 (3.65)	51 (8.54)		
Other Hispanic	132 (3.84)	99 (3.36)	33 (6.11)		
Non-Hispanic White	798 (76.73)	655 (77.94)	143 (71.02)		
Non-Hispanic Black	296 (9.81)	250 (9.99)	46 (8.96)		
Other Race	74 (5.11)	57 (5.05)	17 (5.38)		
Marital status, n (%)				χ^2^ = 2.108	0.349
Married	1229 (85.61)	999 (86.23)	230 (82.71)		
Single	156 (9.78)	109 (9.19)	47 (12.57)		
Unknown	63 (4.61)	50 (4.58)	13 (4.72)		
PIR, n (%)				χ^2^ = 47.362	< 0.001
≤1	299 (14.24)	197 (10.93)	102 (29.78)		
>1	1044 (80.28)	881 (84.01)	163 (62.79)		
Unknown	105 (5.48)	80 (5.06)	25 (7.43)		
Drinking, n (%)	1012 (73.26)	816 (73.85)	196 (70.47)	χ^2^ = 0.956	0.328
Smoking, n (%)				χ^2^ = 36.569	< 0.001
Never smoker	660 (44.25)	548 (45.86)	112 (36.71)		
Former smoker	480 (35.60)	406 (37.68)	74 (25.85)		
Current smoker	308 (20.15)	204 (16.47)	104 (37.44)		
Physical activity, MET·min/week, n (%)				χ^2^ = 2.479	0.115
≤450	909 (59.39)	708 (58.19)	201 (65.06)		
>450	539 (40.61)	450 (41.82)	89 (34.94)		
BMI, kg/m^2^, n (%)				χ^2^ = 2.700	0.259
<25	291 (22.72)	235 (22.68)	56 (22.87)		
25–30	469 (32.13)	382 (33.23)	87 (26.94)		
>30	688 (45.16)	541 (44.09)	147 (50.19)		
Hypertension, n (%)	1129 (72.82)	891 (71.96)	238 (76.88)	χ^2^ = 1.512	0.219
Diabetes, n (%)	410 (20.84)	310 (19.75)	100 (25.98)	χ^2^ = 4.023	0.045
Dyslipidemia, n (%)	1130 (79.46)	913 (80.49)	217 (74.62)	χ^2^ = 2.204	0.138
CVD, n (%)	589 (34.67)	456 (33.10)	133 (42.08)	χ^2^ = 3.571	0.059
Antirheumatics, n (%)	81 (6.38)	65 (6.65)	16 (5.11)	χ^2^ = 0.333	0.564
Glucocorticoids, n (%)	86 (6.13)	60 (5.87)	26 (7.35)	χ^2^ = 0.547	0.460
Nonsteroidal anti-inflammatory agents, n (%)	187 (12.64)	142 (11.84)	45 (16.39)	χ^2^ = 2.941	0.086
Immunologic agents, n (%)	66 (5.37)	54 (5.55)	12 (4.52)	χ^2^ = 0.164	0.685
Antidepressants, n (%)	347 (25.56)	237 (22.45)	110 (40.16)	χ^2^ = 22.135	< 0.001
Antipsychotics, n (%)	30 (1.62)	16 (1.17)	14 (3.73)	χ^2^ = 8.788	0.003
Serum cotinine, ng/mL, Mean (S.E)	61.26 (4.80)	53.87 (4.67)	96.00 (10.16)	t = 0.01	< 0.001
WBC, 1000 cells/uL, Mean (S.E)	7.34 (0.08)	7.23 (0.09)	7.87 (0.20)	t = 0.01	0.005
Lymphocyte count, 1000 cells/uL, Mean (S.E)	2.13 (0.03)	2.11 (0.04)	2.22 (0.07)	t = 0.01	0.245
Segmented neutrophils count, 1000 cell/uL, Mean (S.E)	4.39 (0.05)	4.30 (0.06)	4.81 (0.15)	t = 0.01	0.003
Energy, kcal, Mean (S.E)	1970.13 (29.56)	1986.79 (32.39)	1891.81 (67.77)	t = 1.25	0.212
Mean (S.E)	4.90 (0.07)	5.01 (0.08)	4.39 (0.15)	t = 3.53	< 0.001
Med score, n (%)				χ^2^ = 19.725	< 0.001
<2.82	161 (12.19)	123 (11.85)	38 (13.83)		
2.82–4.30	459 (32.26)	351 (31.00)	108 (38.17)		
4.30–5.87	269 (18.39)	208 (16.97)	61 (25.04)		
≥5.87	559 (37.16)	476 (40.18)	83 (22.96)		

χ^2^: chi-square test; t: t-test; S.E: standard error

PIR: poverty-to-income ratio; BMI: body mass index; CVD: cerebrovascular disease; WBC: white blood cell; MET: metabolic equivalent task; Med: Mediterranean diet

### Association between aMED and depression among RA and non-RA populations

Tables 2 and 3 show the relationship between aMED score and depression in RA and non-RA populations. There was no statistically significant association between aMED and depression among non-RA patients (*P* > 0.05). In RA patients, after adjusted race, smoking, PIR, diabetes, antipsychotics, antidepressants, serum cotinine and neutrophils count (model II), compared with the aMED score of < 2.82 group, the aMED score > 5.87 group was associated with the lower odds of depression (OR = 0.53, 95%CI: 0.29–0.97).

**Table 2:** Association between the aMED and depression in RA patients

Variable	Model I		Model II	
	OR (95%CI)	*P*	OR (95%CI)	*P*
**aMED score**				
< 2.82	Ref		Ref	
2.82–4.30	1.06 (0.60–1.84)	0.849	1.02 (0.59–1.78)	0.933
4.30–5.87	1.26 (0.70–2.30)	0.439	1.32 (0.70–2.49)	0.386
≥ 5.87	0.49 (0.27–0.89)	0.019	0.53 (0.29–0.97)	0.040

Ref: reference; OR: odds ratio; CI: confidence interval

RA: rheumatoid arthritis; aMED: adherence to Mediterranean diet

Model I: crude model

Model II: adjusted race, smoking, PIR, diabetes, antipsychotics, antidepressants, cotinine and neutrophils count

**Table 3:** Association between the aMED and depression in non-RA subjects

Variables	Model 1		Model 2	
	OR (95% CI)	*P*	OR (95% CI)	*P*
Med score	0.96 (0.90–1.02)	0.156	1.00 (0.93–1.07)	0.974
**Med score**				
<2.82	Ref		Ref	
2.82–4.30	1.33 (0.87–2.02)	0.184	1.47 (0.93–2.32)	0.096
4.30–5.87	1.32 (0.85–2.05)	0.223	1.43 (0.86–2.40)	0.170
≥5.87	0.98 (0.61–1.59)	0.933	1.30 (0.75–2.25)	0.356

Ref: reference; OR: odds ratio; CI: confidence interval

RA: rheumatoid arthritis; aMED: adherence to Mediterranean diet

Model I: crude model

Model II: adjusted race, smoking, PIR, diabetes, antipsychotics, antidepressants, cotinine and neutrophils count

### Association between the aMED components and depression

**Fig. 2:**
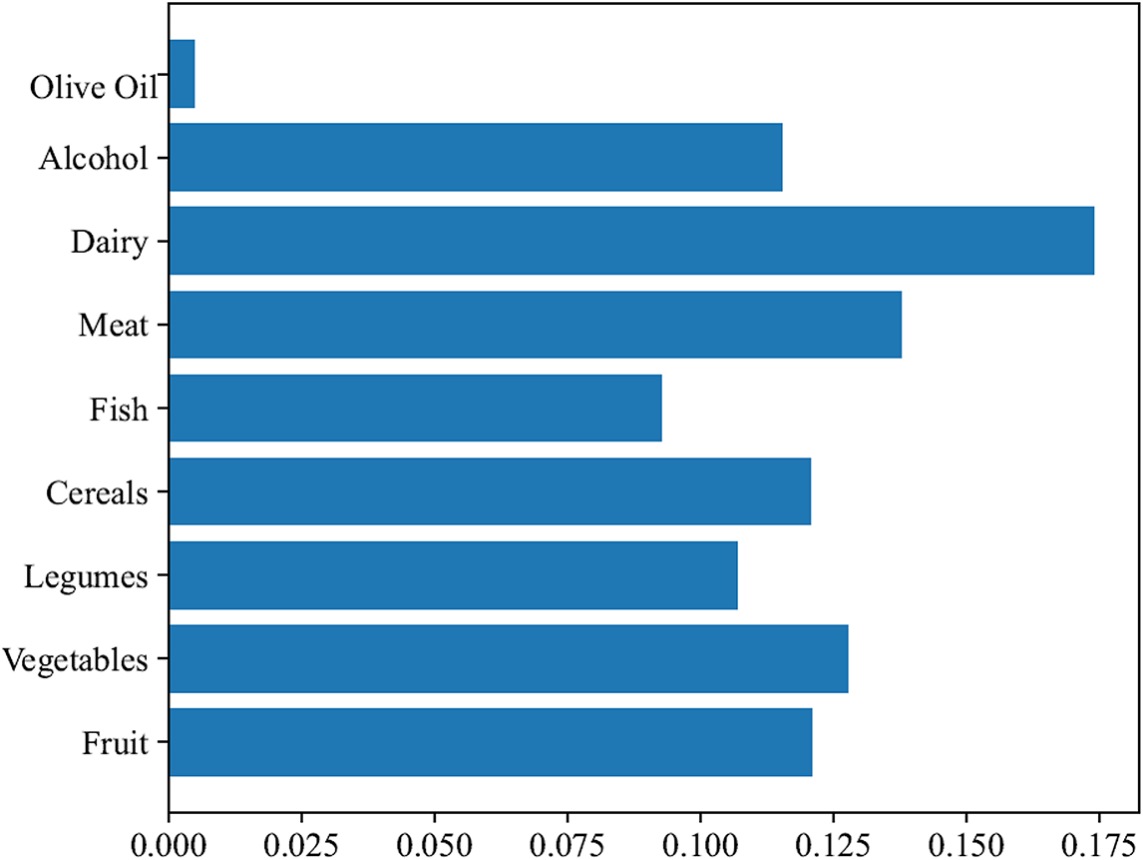
Association between the aMED components and depression

Table 4; Fig. 2 show the association between the main categories of MED and depression. After adjustment for race, smoking, PIR, diabetes, antipsychotics, antidepressants, serum cotinine and neutrophils count in model II, compared with the aMED score < 1 group, higher vegetables (OR = 0.54, 95%CI: 0.34–0.84) and cereals (OR = 0.63, 95%CI: 0.39–0.99) intakes were associated with the lower odds of depression, respectively (*P* < 0.05).

**Table 4:** Association between aMED components and depression in RA patients

Variables	Model I		Model II	
	OR (95%CI)	*P*	OR (95%CI)	*P*
**aMED score-Fruit, cup equivalent**				
< 1	Ref		Ref	
1–2	0.57 (0.35–0.93)	0.026	0.64 (0.39–1.04)	0.070
> 2	0.94 (0.52–1.67)	0.819	1.04 (0.54-2.00)	0.898
**aMED score-Vegetables, cup equivalent**				
< 0.5	Ref		Ref	
0.5-1	0.70 (0.39–1.25)	0.227	0.76 (0.41–1.40)	0.376
> 1	0.49 (0.32–0.76)	0.002	0.54 (0.34–0.84)	0.006
**aMED score-Legumes, ounce**				
< 1	Ref		Ref	
1–2	1.46 (0.69–3.09)	0.314	1.35 (0.60–3.02)	0.469
> 2	0.79 (0.43–1.48)	0.466	0.71 (0.37–1.36)	0.301
**aMED score-Cereals, ounce**				
< 1	Ref		Ref	
1–1.5	0.58 (0.35–0.99)	0.044	0.61 (0.34–1.07)	0.081
> 1.5	0.58 (0.37–0.91)	0.019	0.63 (0.39–0.99)	0.049
**aMED score-Fish, ounce**				
< 1	Ref		Ref	
1–2.5	0.69 (0.29–1.66)	0.402	0.76 (0.30–1.91)	0.550
> 2.5	0.55 (0.27–1.10)	0.090	0.59 (0.28–1.23)	0.157
**aMED score-Meat, ounce**				
< 1	Ref		Ref	
1–1.5	0.96 (0.37–2.46)	0.925	1.07 (0.40–2.87)	0.897
> 1.5	0.81 (0.60–1.08)	0.151	0.81 (0.59–1.11)	0.190
**aMED score-Dairy, cup equivalent**				
< 1	Ref		Ref	
1–1.5	1.04 (0.60–1.79)	0.901	0.98 (0.56–1.70)	0.940
> 1.5	1.19 (0.83–1.72)	0.345	1.12 (0.76–1.65)	0.567
**aMED score-Alcohol, gram**				
> 24	Ref		Ref	
< 12	2.07 (1.00-4.31)	0.051	1.89 (0.90–3.96)	0.093
12–24	1.57 (0.50–4.91)	0.433	1.70 (0.51–5.66)	0.382
**aMED score-Olive oil, gram**				
< 14	Ref		Ref	
14–28	–	< 0.001	–	< 0.001
> 28	–	< 0.001	–	< 0.001
Med score-Olive oil, gram	0.40 (0.37–0.44)	< 0.001	0.38 (0.34–0.42)	< 0.001

Ref: reference; OR: odds ratio; CI: confidence interval

RA: rheumatoid arthritis; aMED: adherence to Mediterranean diet

Model I: crude model

Model II: adjusted race, smoking, PIR, diabetes, antipsychotics, antidepressants, cotinine and neutrophils count

## Discussion

In the present study, we found that greater aMED, especially high intake of vegetables and cereals, were associated with the lower odds of depression in RA populations.

Although great progress has been made in the prevention and treatment of RA over the past half century, the pathogenesis of RA, a highly teratogenic autoimmune disease, has not been fully understood [25–27]. Patients with RA suffer from joint distortions and loss of physical function, which in turn leads to large fluctuations in social and psychological functioning [28, 29]. An important factor contributing to poor prognosis for patients with RA is psychiatric disorders, of which mood disorders such as depression are the most common. There is a close two-way relationship between RA and depression, and several studies have shown that the release of a large number of cytokines by immune dysfunction in RA patients plays an important role in the onset and progression of depression [30–32]. In the RA process, activated T cells can stimulate macrophages to produce a large number of inflammatory factors such as tumor necrosis factor-α (TNF-α), promote excessive multiplication of synovial cells, and then damage bone and joints, and also promote the production of rheumatoid factors and fractalkine [33].

Most chronic non-infectious disease can be prevented with a healthy diet and lifestyle, although the best approach remains controversial. Evidence accumulating in the general population suggests that some traditional dietary patterns are associated with health benefits differently, such as the Med and the vegetarian diet. The MED is the dietary pattern first determined by KEYS A et al. [34] in the 60s of the 20th century, mainly characterized by fresh fruits, vegetables, natural grains, legumes, fish and extra virgin olive oil, while ensuring the intake of eggs, meat and dairy products, reducing the intake of refined sugar and red meat, and nowadays, herbs are also used in large quantities [35–37]. Compared to the usual diet, the MED is effective in reducing the weight of people with diabetes and metabolic syndrome, as well as in postpartum women, preventing the development of cardiovascular diseases (CVD) and certain neurodegenerative diseases and cancer, although the results are sometimes less significant [38]. The beneficial role of the MED in the prevention of CVD has been widely concerned, although moderate to high levels of inconsistency have been reported. Results from one randomized controlled trial suggest that the MED (crude rate per 1000 person-years: 28.1 [95%CI: 27.9–28.3]) was superior to low-fat diets 37.7 (95%CI: 37.5–37.9) in preventing major CVD events in secondary prevention, and the benefit is more pronounced in men [39]. As an inflammatory autoimmune disease, the clinical prevention and treatment of RA has benefited from the MED. One study showed that patients with RA did experience a reduction in inflammatory activity in the body, lower disease activity score in 28 joints (DAS28) and health assessment questionnaire (HAQ) scores, and modest improvements in physical ability and activity by adopting a MED [40, 41], which was consistent with our results. Similarly, Sköldstam et al. [41] pointed out MED patterns and the use of fish oil supplements appear to be promising to reduce the risk of inflammation and comorbidities in populations with RA. In addition, a population-based case-control study showed a 21% reduction in the incidence of RA in people with high aMED scores (OR = 0.79, 95%CI: 0.65–0.96) and lower OR values in men (OR = 0.49, 95%CI: 0.33–0.73) in people with low aMED scores, but there was no significant association in women [42]. Although high aMED scores are not always so significant in studies of patients with RA, there is reason to speculate that some models of intervention regarding the MED may be potentially beneficial in the treatment of patients with RA.

Based on the above findings, we explored the relationship between aMED and the risk of depression in people with RA. Present study suggested that greater aMED was associated with the lower odds of depression in RA patients. Our findings further support the importance of the MED as a potentially healthy eating pattern. The MED is a dietary pattern consisting of multiple components, each of which is also strongly focused on the link between the risk of developing disease. The MED advocates increased intake of fish, fruits and vegetables, as these foods are rich in many elements that are beneficial for depression (such as calcium, magnesium, iron, vitamin C, etc.). Our study results were consistent with the conclusion of previous studies, among the various components of the MED, higher intake of vegetables and cereals in the prevention of the risk of depression in people with RA needs to be considered. The prevention and treatment effect of fresh vegetables on the occurrence and development of RA and depression may be due to its beneficial biological effects such as antioxidant, anti-inflammatory, and antimutagenic. Vegetables are rich in dietary fiber and vitamins, which can play an important role in regulating steroid hormone concentration and metabolism, activating antioxidant mechanisms, regulating detoxification enzymes and stimulating the immune system. A recent study has also shown that a certain amount of vegetables and fish intake has a significant improvement in clinical levels of depression in young and older adults [43–45]. Similarly, a longitudinal study in the UK on the relationship between vegetable consumption and well-being showed that increasing vegetable intake can ensure good health in the long run and also benefit peoples’ mental health in the short term [46].

Many previous studies of anti-inflammatory dietary interventions have shown potential benefits for increasing cereal intake in the prevention and treatment of depression and RA. Study suggested that using cereals in breakfast can reduce the risk of RA [47]. A study on the relationship between food and perceived stress and depressive symptoms among UK university students showed that eating healthy foods (e.g. salads, cooked vegetables, whole grain cereals) was significantly negatively associated with perceived stress and depressive symptom scores. Interventions to reduce depressive symptoms and stress in students may also lead to healthier food consumption [48]. In addition, a review of building a food pyramid for people with rheumatoid arthritis to reduce inflammatory effects suggests that gluten-free whole grains are beneficial as a source of primary carbohydrates in the consumption of carbohydrates, which are essential in the human diet [49]. Previous studies have shown that dietary fiber has beneficial effects on the regulation of intestinal flora and can improve the inflammatory state of the whole body, negatively correlated with highly sensitive C-reactive protein, interleukin-6 and tumor necrosis factor-α receptor-2 [50]. However, different types of cereals have different or even opposite effects on the elimination of RA inflammation. For example, the high fiber and low monosaccharides in natural oats, bran, or unrefined grains have an inhibitory effect on RA inflammation, and eating highly processed grains is likely to achieve the opposite effect. Therefore, although evidence suggests that cereals are strongly associated with RA populations and depression, the link needs to be carefully explored.

The above evidence suggest that the MED may be a very effective adjunct to RA with depression. Maintaining a high aMED has potential benefits for the prevention and treatment of this population. A representative and high-quality NHANES database was used, and the regression models were adjusted considering the covariates. Several limitations need caution in interpreting our findings. First, despite the 24-h dietary recall interview was the valid method to obtain the dietary intake data, participants’ memory bias may pose a challenge to acquiring the accurate evaluation, especially for the elderly included in this study. Moreover, in the multivariate logistic regression model, as many covariates as possible were included, but the confounding effect of missing or unknown factors still cannot be excluded. Finally, the cross-sectional study design of this study could not establish a causal relationship between the aMED and depression among RA patients.

## Conclusion

In conclusion, we found that greater aMED was associated with the lower odds of depression in RA patients. Thus, our results provide evidence that greater adherence to MED may improve mental health in adults. To elucidate whether true causal associations exist between diet and depression, further more rigorous cohort studies are needed.

### Electronic supplementary material

Below is the link to the electronic supplementary material.

Supplementary Material 1

## Data Availability

Publicly available datasets were analyzed in this study. This data can be found here NHANES, NHANES Questionnaires, Datasets, and Related Documentation (cdc.gov).

## Abbreviations

RA: Rheumatoid arthritis
aMED: Adherence of Mediterranean dietary
MED: Mediterranean diet
NHANES: National Health and Nutrition Examination Survey
NCHS: National Centers for Health Statistics
CDC: Disease Control and Prevention
WBC: White blood cell
BMI: Body mass index
PHQ-9: Patient Health Questionnaire Depression Scale
MEC: Mobile Examination Center
USDA: United States Department of Agriculture
PIR: Poverty-to-income ratio
MCQ: Medical condition questionnaire
HbAlc: Hemoglobin A1C
SBP: Systolic blood pressure
DBP: Diastolic blood pressure
TC: Total cholesterol
HDL-C: High density lipoprotein cholesterol
MET: Metabolic equivalent task
PSU: Pseudo-primary sampling unit
CI: Confidence interval
S.E.: Standard error
ORs: Odds ratios
