# Supplementary material for: Association between the adherence to Mediterranean diet and depression in rheumatoid arthritis patients: a cross-sectional study from the NHANES database
Source: J Health Popul Nutr. 2024 Jul 5;43:103. doi: 10.1186/s41043-024-00572-w (PMC11227153; doi:10.1186/s41043-024-00572-w)
Supplement: Supplementary file 1 — Supplementary Material 1 [file 41043_2024_572_MOESM1_ESM.docx]

Table S1 Univariate logistics regression analysis of depression in RA patients

| Variables | OR (95%CI) | *P* |
| --- | --- | --- |
| Age, years | 0.99 (0.98-1.00) | 0.071 |
| Gender |  |  |
| Male | Ref |  |
| Female | 1.36 (0.88-2.11) | 0.167 |
| Race/Ethnicity-Recode |  |  |
| Mexican American | Ref |  |
| Other Hispanic | 0.78 (0.44-1.36) | 0.376 |
| Non-Hispanic White | 0.39 (0.26-0.57) | <0.001 |
| Non-Hispanic Black | 0.38 (0.24-0.61) | <0.001 |
| Other Race | 0.46 (0.20-1.05) | 0.065 |
| Marital status |  |  |
| Married | Ref |  |
| Single | 1.43 (0.88-2.31) | 0.147 |
| Unknown | 1.07 (0.50-2.30) | 0.854 |
| Family PIR |  |  |
| ≤1 | Ref |  |
| >1 | 0.27 (0.18-0.43) | <0.001 |
| Unknown | 0.54 (0.30-0.96) | 0.035 |
| Drinking |  |  |
| No | Ref |  |
| Yes | 0.84 (0.60-1.19) | 0.332 |
| Smoke |  |  |
| Never smoker | Ref |  |
| Former smoker | 0.86 (0.54-1.36) | 0.512 |
| Current smoker | 2.84 (1.91-4.23) | <0.001 |
| Physical activity, MET·min/week |  |  |
| ≤450 | Ref |  |
| >450 | 0.75 (0.52-1.08) | 0.119 |
| BMI, kg/m^2^ |  |  |
| <25 | Ref |  |
| ≥25 and ≤29.9 | 0.80 (0.48-1.34) | 0.402 |
| >29.9 | 1.13 (0.70-1.82) | 0.614 |
| Hypertension |  |  |
| No | Ref |  |
| Yes | 1.30 (0.85-1.97) | 0.224 |
| Diabetes |  |  |
| No | Ref |  |
| Yes | 1.43 (1.01-2.03) | 0.049 |
| Dyslipidemia |  |  |
| No | Ref |  |
| Yes | 0.71 (0.45-1.12) | 0.141 |
| CVD |  |  |
| No | Ref |  |
| Yes | 1.47 (0.98-2.20) | 0.063 |
| Antirheumatics, n(%) |  |  |
| No | Ref |  |
| Yes | 0.76 (0.29-1.98) | 0.567 |
| Adrenal cortical steroids, n(%) |  |  |
| No | Ref |  |
| Yes | 1.27 (0.66-2.44) | 0.462 |
| Nonsteroidal anti-inflammatory agents, n(%) |  |  |
| No | Ref |  |
| Yes | 1.46 (0.94-2.27) | 0.093 |
| Immunologic agents, n(%) |  |  |
| No | Ref |  |
| Yes | 0.81 (0.28-2.33) | 0.687 |
| Antidepressants, n(%) |  |  |
| No | Ref |  |
| Yes | 2.32 (1.61-3.34) | <0.001 |
| Antipsychotics, n(%) |  |  |
| No | Ref |  |
| Yes | 3.28 (1.41-7.62) | 0.006 |
| Cotinine, ng/mL | 1.01 (1.01-1.01) | <0.001 |
| WBC, 1000 cells/uL | 1.10 (0.99-1.21) | 0.075 |
| Lymphocyte number, 1000 cells/uL | 1.03 (0.96-1.11) | 0.383 |
| Segmented neutrophils num, 1000 cell/uL | 1.17 (1.06-1.29) | 0.002 |
| Energy intake, kcal | 1.00 (1.00-1.00) | 0.227 |

OR: odds ratio; CI: confidence interval; RA: rheumatoid arthritis; PIR: poverty to income ratio; MET: metabolic equivalent task; BMI: body mass index; WBC: white blood cell.

Table S2 Incidence of depression in RA patients with different aMED scores

| Variables | Total (N=1448) | aMED score | | | | Statistics | *P* |
| --- | --- | --- | --- | --- | --- | --- | --- |
|  |  | <2.82 (N=161) | 2.82-4.30 (N=459) | 4.30-5.87 (N=269) | ≥5.87 (N=559) |  |  |
| Depression, n (%) |  |  |  |  |  | χ² = 6.350 | <0.001 |
| No | 1158 (82.46) | 123 (80.11) | 351 (79.24) | 208 (76.11) | 476 (89.17) |  |  |
| Yes | 290 (17.54) | 38 (19.89) | 108 (20.76) | 61 (23.89) | 83 (10.83) |  |  |

χ²: Rao-Scott Chi-square test; aMED: adherence to Mediterranean diet; RA: rheumatoid arthritis.

Table S3 Differences between groups of different aMED scores

| Variables | *P* | P1 |
| --- | --- | --- |
| Different groups |  |  |
| <2.82: 2.82-4.30 | 1.000 | 1 |
| <2.82: 4.30-5.87 | 1.000 | 1 |
| <2.82: ≥5.87 | 0.025 | 0.025 |
| 2.82-4.30: 4.30-5.87 | 1.000 | 1 |
| 2.82-4.30: ≥5.87 | 0.003 | 0.00337 |
| 4.30-5.87: ≥5.87 | 0.022 | 0.0217 |

aMED: adherence to Mediterranean diet.
